# Antiviral Strategies Using Natural Source-Derived Sulfated Polysaccharides in the Light of the COVID-19 Pandemic and Major Human Pathogenic Viruses

**DOI:** 10.3390/v14010035

**Published:** 2021-12-24

**Authors:** Bimalendu Ray, Imran Ali, Subrata Jana, Shuvam Mukherjee, Saikat Pal, Sayani Ray, Martin Schütz, Manfred Marschall

**Affiliations:** 1 Department of Chemistry, The University of Burdwan, Burdwan 713104, West Bengal, India; imran.ia.ali@gmail.com (I.A.); subratajanachem@gmail.com (S.J.); shuvamm82@gmail.com (S.M.); pal.saikat87@gmail.com (S.P.); 2 Institute for Clinical and Molecular Virology, Friedrich-Alexander University (FAU) of Erlangen-Nürnberg, 91054 Erlangen, Germany

**Keywords:** sulfated polysaccharides, antiviral activities and mechanisms, drug structure-activity relationship, antiviral efficacy, heparin mimetics, in vivo studies, virus entry as a target, major human pathogenic viruses, emerging viral infections

## Abstract

Only a mere fraction of the huge variety of human pathogenic viruses can be targeted by the currently available spectrum of antiviral drugs. The severe acute respiratory syndrome coronavirus 2 (SARS-CoV-2) outbreak has highlighted the urgent need for molecules that can be deployed quickly to treat novel, developing or re-emerging viral infections. Sulfated polysaccharides are found on the surfaces of both the susceptible host cells and the majority of human viruses, and thus can play an important role during viral infection. Such polysaccharides widely occurring in natural sources, specifically those converted into sulfated varieties, have already proved to possess a high level and sometimes also broad-spectrum antiviral activity. This antiviral potency can be determined through multifold molecular pathways, which in many cases have low profiles of cytotoxicity. Consequently, several new polysaccharide-derived drugs are currently being investigated in clinical settings. We reviewed the present status of research on sulfated polysaccharide-based antiviral agents, their structural characteristics, structure–activity relationships, and the potential of clinical application. Furthermore, the molecular mechanisms of sulfated polysaccharides involved in viral infection or in antiviral activity, respectively, are discussed, together with a focus on the emerging methodology contributing to polysaccharide-based drug development.

## 1. Introduction

Viruses represent opportunistic, replicative units, tightly integrated into the regulatory machinery of their infected host cells and can be found in the entire sphere of living organisms. Virus infections have a huge impact on life on this globe and are highly complex in their way of virus–host interaction, whereas the viral genetic composition varies substantially between different viruses. For example, the Ebola virus encodes only seven major proteins but, nevertheless, can have a significant impact on the life of infected populations [1]. Other viruses, termed as eukaryotic giant viruses can have extra genes for encoding proteins active in metabolic processes, otherwise typically found in living organisms [2]. According to the complexity of virus regulation, a number of targeting options can be considered for the conceptualization of antiviral drugs, which may target the entry, replication, proteolytic processing and particle egress steps of the infectious virus cycle [3]. Antiviral drug approaches still have a rate-limiting issue that only a very low number of compounds are available to combat more than 220 human virus infections known. Furthermore, just a selected repertoire of antiviral drugs are formally or provisionally approved for medical treatment [3]. A rapidly growing human population and the simultaneous landscape change in the last century have led to an increase of infectious viruses from wildlife. Especially viruses originating from domesticated species, primates and bats which frequently acquire the capacity to infect and to spread among humans, thus spilling over from other geographic regions and/or from the animal kingdom [4,5]. Human coronaviruses, such as SARS-CoV, Middle East respiratory syndrome coronavirus (MERS-CoV) and now SARS-CoV-2, are leading examples of rapidly emerging viruses for which no particular treatments have been available before. It goes without saying that in a situation like the COVID-19 pandemic, resulting from the human-to-human spread of SARS-CoV-2 infections, all options of antiviral drug development, vaccine production and preventive measures are intensely examined towards a game-changing combination of interventions. However, in specific cases, the time schedules of development represent restricting factors and thus need to be addressed by forward-planning research. One example of a widely available and broadly bioactive group of compounds are polysaccharides, particularly those omnipresent in natural environments and produced by living organisms, such as microorganisms, plants and the marine biotope. Generally, natural products derived from both marine and land biota are a valuable source of front-line drug development [6,7,8,9,10,11,12,13]. They may even outperform synthetic screening libraries in terms of structural diversity and biological relevance [8,14,15]. Natural polymers, specifically polysaccharides, on account of their inherent unique properties as well as their attractive biological activities are of great current interest for biomedical applications. The properties mainly favoring the aspects of their use as antiviral candidate compounds comprise their limited polysaccharide-induced toxicity, biocompatibility and biodegradability [16,17,18,19,20,21,22]. In particular, the astonishing diversity of sulfated polysaccharides from marine and plant biota are prospective bioactive chemicals [12,13,23,24]. Contrary to their animal counterparts, sulfated polysaccharides of marine origin are considered to be safe and non-immunogenic in many cases [25,26,27]. Emerging evidence demonstrated that sulfated polysaccharides offer exciting pharmacological perspectives for the generation of antiviral drugs [10,18,23,24,28,29,30,31]. Importantly, the mode of action of these polymers is mostly different from clinically used antiviral drugs [10,24,29]. Moreover, by virtue of their structural uniqueness and high molecular weight (MW), sulfated polysaccharides have characteristics that small drug molecules do not have. For instance, the pharmacodynamics and pharmacokinetics of these polymers can be adjusted by the fine-tuning of their molecular weight and structural characteristics [17,32,33]. This adjustability has been considerably investigated in the context of cancer therapy, an area in which the generation of polysaccharide-based carriers of drug delivery has become an ongoing focus of research activities [34,35,36]. Incidentally, multivalency plays a major role in biological processes and particularly in the relationship between pathogenic microorganisms and their host that involves protein–polysaccharide interaction [37]. Sulfated polysaccharides are multivalent, meaning that many structural components of the backbone or pendant chains can simultaneously bind to more than one complementary binding protein or receptor that are present on the targets such as cellular surfaces. As several individual ligand–receptor bonds work together, multivalent interactions are usually stronger than monovalent interactions. In particular, semisynthetic sulfated polysaccharides can imitate such type of multivalent interaction that is common in biological systems including virus–host cell interaction [38].

As one particular example for the relevance of polysaccharide molecules in human virus infections, the increased rate of thromboembolic events in COVID-19 patients should be mentioned that shows that coagulopathy plays a role in the pathophysiology of SARS-CoV-2 [39]. The use of low-molecular-weight heparin (LMWH) decreases mortality in patients with severe coronavirus coagulopathy, according to new findings [40]. Although the entire spectrum of heparin’s positive impacts for COVID-19 patients is still being investigated, promising clinical results already suggest that heparin-mimicking compounds might be beneficial for the treatment or prevention of SARS-CoV-2 infections [41]. Beyond SARS-CoV-2, a major focus of current research is to further resolve the diverse biological effects of sulfated polysaccharides, especially to investigate structure–activity relationships. Limited access to pure sulfated polysaccharides with established structural features and MW is a visible problem in exploring the variety of biological effects. Yet, these structural features are decisive in determining the biological and/or therapeutic capabilities of individual prototypes of this group of polymers. Moreover, although polysaccharides are the most common components of the plant world, their sulfated derivatives are only biosynthesized by seaweeds and mammals [42]. Thus, polysaccharide sulfates, which are most potent in producing strong biological effects such as antiviral activities, are currently generated on a biotechnological basis by using a two-step process, i.e., an initial extraction of the polysaccharide mass from plant material followed by a chemical sulfation reaction towards an oligosulfated entity of individual polysaccharide determinants. A recently proposed cost-effective single-stage process has the ability to generate a large number of sulfated polysaccharides with different structural features from plant materials, and thus inducing potential biological activities including antiviral activity in the final product [43]. Along with the alteration of the typical hydroxyl groups into sulfates, a functionality that is rarely found in higher plants, this method also changes some of the properties (like the MW, composition, sulfate content and others) of the generated sulfated polysaccharides, and, therefore, the chances of producing libraries of such polymers with interesting biomolecular properties can be increased [43,44,45,46,47].

From this point of view, we focus on sulfated polysaccharides that show antiviral effects on their own. In this review, the initial part presents a historical view and an overview of the sulfated polysaccharide-based antiviral agents. Herein, we describe the structural features of several families of naturally occurring sulfated polysaccharides by analyzing their antiviral activities. The next focus is given to polysaccharides that have been chemically sulfated, and to chemically sulfated polysaccharides produced by a one-step extraction-sulfation method. Incidentally, the synthesis of new molecules possessing diverse structures utilizing this cost-effective one-step will be a useful addition to the arsenal of antivirals. Additionally, these sulfated polysaccharides will help to establish an improved understanding of the structure–activity relationship (SAR). Then, the mode of action of these sulfated polysaccharides and their current analysis in pre-clinical and clinical studies is described. As this review does not go into detail with the synthesis or biological activities of other types of sulfated polymers, such as sulfated non-carbohydrates, it should be emphasized that these issues have already been discussed elsewhere [17,48,49,50]. Finally, the present review provides a comprehensive analysis of sulfated polysaccharide-based antiviral agents. Furthermore, it provides an update-insight into the SAR of sulfated polysaccharides, their mechanism of action and the future perspective of in vivo studies, with a specific focus on developments in the past, pandemic-imprinted months and years.

## 2. The Origins and Early Steps of Natural Source-Based Antiviral Drug Development

In 1947, the first report of the antiviral activity of polysaccharides appeared, under mostly serendipitous circumstances [51], as the authors were still far away from our current knowledge about viruses and their targeting by inhibitory molecules. Then in 1947 and 1948, researchers quickly investigated specific polysaccharides for their antiviral efficacies against influenza and mumps viruses [52,53]. After these initial periods, Gerber and co-workers observed inhibitory effects towards mumps virus and influenza B virus exerted by marine algae-derived polysaccharides [54]. However, these findings drew little attention because their antiviral activities were thought to be essentially nonspecific. Later on, Ehresmann and co-workers (1977) causally linked polysaccharide-containing fractions of red algae extracts with the suppressive effects on the replication of herpes simplex and other viruses [55], before Richards and co-workers (1978) reported similar findings [56]. In 1987, Nakashima et al., found that sulfated polysaccharides from *Schizymenia pacifica*, a red alga, possess the potential to inhibit the reverse transcriptase activity of human immunodeficiency virus type 1 (HIV-1) [57,58].

Meanwhile, the polyanionic property of polysaccharide compounds was considered to be generally important for antiviral activity [59,60]. Especially, the potential of sulfated polysaccharides, such as dextran sulfate, heparin, and agar, were evaluated as inhibitors of viral replication in vitro [59,60,61,62]. To explain how polyanionic compounds may exert inhibitory effects on viruses, two theories have been established. The first involved a viral adsorption inhibition mechanism, in which the polymer would adhere to the surface of infectious virions and subsequently prevents the host cell attachment [61,63]. A second, alternative point of view favored the fact that polyanions could boost the cellular interferon production, a signal transduction system induced by virus-infected cells to notify adjacent cells in order to generate a largely antiviral intracellular environment [17,64,65,66]. There was, however, no clear consensus on which mechanism was preferred or how a polysaccharide compound might act in one or more antiviral modes of action. Then, during the further study of the antiviral activity of sulfated polysaccharides, three primary investigative stages arose. First, naturally occurring sulfated polysaccharides are being studied in detail until today [10,29,30,31,67,68,69]. The second important stage of advances occurred in 1987, when Ito and co-workers chemically sulfated a bacterial polysaccharide progenitor to dextran sulfate, and subsequently blocked the replication of HIV-1 in vitro [70]. Finally, as illustrated by Table 1, a list of diverse forms of sulfated polysaccharides, basically all of which are occurring in natural sources, is provided and the information is added in which way antiviral activities have been determined for a variety of viruses. In 2015, a new one-stage strategy combining polysaccharide extraction and chemical sulfation emerged [43].

## 3. Naturally Occurring Sulfated Polysaccharides-Based Antivirals

### 3.1. Seaweed-Derived Compounds

Seaweeds, including brown (Phaeophyceae), green (Chlorophyta), and red (Rhodophyta), biosynthesize various sulfated polysaccharides as a key component of their cell walls [116,117,118,119]. The structures of these polymers vary greatly, and many of them exhibited a wide spectrum of antiviral activity [29,31,67,120,121]. The following section will explore a number of promising naturally occurring sulfated polysaccharides analysing the antiviral activity of these polymers.

#### 3.1.1. Fucoidans

Fucoidan polysaccharides containing significant percentages of L-fucose and sulfated ester groups are constituents of brown algae and some marine invertebrates [122]. Conchie and Percival (1950) depicted fucoidan from the brown algae *Fucus vesiculosus* as a polysaccharide-based on L-fucose with mainly α-(1,2) glycosidic bonds and sulfate groups at position 4 [123]. In 1993, Patankar and co-workers reinvestigated the structure of fucoidan of this alga and it was shown that the main chain of this polysaccharide contains (1,3)-linked Fuc*p* residues [124]. More recent studies showed that the backbone of fucoidan is built up of alternating α-(1,3)- and α-(1,4)-linked Fuc*p* residues as displayed in Table 1 [125,126,127]. Later on, Karmakar and co-workers (2009) reported the presence of a fucoidan the core region of which is composed primarily of α-(1,2)- and α-(1,3)-linked Fuc*p* residues with sulfate groups at position 4 and 2 [128]. These complex polysaccharides inhibited a wide variety of viruses including HIV [81,129,130,131], herpes simplex virus (HSV) [76,77,79,80,101,132,133,134,135,136,137,138], Influenza virus (IV) [139,140,141,142,143], avian influenza virus (AIV) [144], human cytomegalovirus (HCMV) [132,134], Newcastle disease virus (NDV) [107,145] bovine viral diarrhoea virus [31,78,146], SARS-CoV-2 [82,147,148,149] and murine norovirus [150]. Notably, several sulfated polysaccharides exert varied inhibitory efficacy against different viruses, implying that the target molecules with which polysaccharides interact are somewhat different (Table 1). Recently, an in vitro assay with two fucoidans revealed that these polymers are effective SARS-CoV-2 inhibitors [148]. In fact, they outperformed remdesivir (RDV), a drug currently licenced for use as an emergency treatment in severe COVID-19 infections [82]. Additionally, the sulfated galactofucan from *Saccharina japonica* showed a strong binding ability to SARS-CoV-2 spike glycoproteins [147].

#### 3.1.2. Galactans

The main polysaccharide components of red algae are sulfated galactans, which usually have a linear backbone built up of alternating 3-linked β-D-Gal*p* and 4-linked α-Gal*p* residues. The latter have the L-configuration in the agar group of polysaccharides, but the D-configuration in carrageenans. In addition, 4-linked residues may be present, in part or completely, as 3,6-anhydro derivatives. This clear-cut separation between carrageenans and agarans has been upset by the finding of a third group, named DL-galactan hybrids, in which the 3,6-anhydro galactose units can have D- and L- configurations in the same molecule (Table 1). Concerning antiviral activity, agarans inhibit herpes simplex virus type 1 (HSV-1) [151], sulfated galactans are effective HSV, HMPV, white spot disease virus inhibitors [85,87,152,153,154,155,156], and DL-hybrid galactan sulfate exerts activity against HSV-1, dengue virus (DENV) [86] and DENV-2 [93]. As of 1987, carrageenans have been found to exert antiviral activity against an array of viruses (Table 1), both enveloped and non-enveloped, including HIV [90], HSV [89,91,157,158,159], human papilloma viruses (HPV) [160], hepatitis-A [161], DENV [89,93,162,163], JEV [104], rhinoviruses (RVs) [164], and tobacco mosaic virus [165], rift valley fever virus [158], measles morbillivirus [105], influenza virus [94,95,166,167], influenza A virus(IAV) [92], bovine herpesvirus type 1 [168], suid herpesvirus type 1 [168], porcine reproductive and respiratory syndrome virus (PRRSV) [169], rabies virus (RABV) [170], SARS-CoV-2 [171], and SARS-CoV-2 [171,172,173,174,175,176,177,178]. For instance, carrageenan isolated from *Meristiella gelidium* has a very high selectivity index (25,000) for herpes simplex virus type 2 (HSV-2), signifying that this biopolymer is a reasonable contender for further antiviral research [89]. Furthermore, it had potent inhibitory effects in vivo against HSV [179,180] and murine cytomegalovirus [181]. Lynch et al., (2021) recently investigated the impact of *Fucus vesiculosus*, *Mastocarpus stellatus*, and algal derivatives (fucoidan and κ-carrageenan) on the performance of the oyster *Crassostrea gigas*, as well as the ostreid herpesvirus-1 microvar (OsHV-1 μVar) and bacteria *Vibrio* spp. development [182]. OsHV-1 μVar prevalence was reported to be much reduced in treated oysters, and κ-carrageenan was found to decrease viral replication (loads), while OsHV-1 μVar was not detected in fucoidan-treated oysters after Day 8 of the 26-day study. From the standpoint of oyster production, the two-fold effect of improving an oyster’s immunological function while lowering OsHV-1 μVar would be extremely helpful to the industry’s long-term viability.

#### 3.1.3. Ulvan

Relating to marine green algal polysaccharides, ulvan is a hot topic of research aimed at developing novel therapeutic agents [183,184]. Typically, this polymer contains Rha, GlcA, IduA, Xyl residues and sulfate [185,186,187,188,189]. The major repeating disaccharide units as shown in Table 1 consist of GlcA and Rha-3-sulfate, and iduronic acid with Rha-3-sulfate [186]. Ulvan, like other sulfated polysaccharides, can prevent virus adsorption and thereby viral entrance into the cell [107,184,190,191]. It has been shown to be effective against a number of viruses including Japanese encephalitis virus (JEV) [192], influenza virus (H1N1) [139], DENV [100], AIV [144], vesicular stomatitis virus [106], measles virus [105], HSV [45,108,134], NDV [107], Indiana vesiculo virus [106], and human metapneumo virus (HMPV) [193]. This sulfated polymer is also useful in managing viruses associated with poultry-linked operations, such as the NDV, a deadly virus that causes large economic losses in hens. In vitro experiments on Vero cells revealed that ulvan has an IC_50_ of 0.1 μg mL^−1^ for inhibiting viral entrance [107]. It prevents the intact protein F0 from being cleaved into the mature form, which inhibits viral fusion. This biopolymer has superior anti-cell-cell fusion effects than fucoidans, and when used together, it can have even stronger effects [107]. Concerning the antiviral efficacy against the avian flu AIV-H9N2, ulvan from *U. pertusa* by itself demonstrated only mild efficacy [144]. Yet, if paired with a vaccine against the same virus, it resulted in a hundred percent increase in antibody titer compared to the immunisation alone. The immunomodulatory activity of the polymer was thought to be accountable for the increased humoral immune response [144]. Even oligosaccharides made from ulvan have substantial antiviral properties. For example, the low MW oligomer (4.3 kDa) derived from the ulvan of *U. pertusa* through chemical degradation shows greater efficacy against Avian Leukosis Virus Subgroup J (ALV-J) than ulvan itself, and this molecule bonds with viral particles and impedes ALV-J adsorption onto the host cells [194]. Reisky and co-workers demonstrated that a marine bacterial enzymatic cascade is able to degrade ulvan yielding oligo- and monosaccharides [195] thereby paving the way for generating low MW sulfated molecules.

#### 3.1.4. Alginic Acids

The majority of brown seaweeds are prospective sources of alginate, a promising biopolymer that can also be produced from a variety of microorganisms. Structurally, alginic acid is a linear polymer made up of β-D-Man*p*A and α-L-Gul*p*A acid residues, with no sulfate ester. These monomers are glycosidically linked at the C-1 and C-4 positions to produce the alginate. The polymer chain has been demonstrated to be made up of three different types of blocks. The M blocks are totally made up of D-ManA (Table 1), the G blocks contain exclusively L-GulA residues (Table 1), and the MG blocks be composed of alternate between D-ManA and L-GulA-derived residues [196,197,198,199]. The alginate-derived therapeutic agent “911” inhibits the viral reverse transcriptase [200] and the viral polymerase [201], thereby exhibiting activity against HIV-1 and HBV. Antiviral activity of alginate polymers was also seen against other viruses such as HSV-1 and HSV-2, as well as the HPV [101,202,203,204]. Sinha et al. (2010) found that chemically sulfated guluronans produced from *Sargassum tenerrimum* are effective inhibitors of HSV type 1 (HSV-1) by imitating the entry receptor’s active domain [74]. Similarly, the anti-HBV activity and mechanism of action of marine-derived polyguluronate sulfate (PGS) in vitro have also been reported [205]. The sulfated alginate from *Sphacelaria indica* and *Laminaria angustata* exhibited anti-HSV-1 activity by inhibiting HSV attachment to cells by direct interaction of polysaccharides with viral particles [76,77]. The crude polysaccharide 375 isolated from the seaweed *Ecklonia kurome* shows good anti-SARS-CoV-2 infection activity in cell culture with EC_50_ values of 27 nM and low toxicity, although the three polysaccharides purified by anion exchange chromatography were less active implying that the cocktail-like polysaccharide worked synergistically by targeting multiple key molecules implicated in the virus infection and replication [206]. For instance, the purified alginic acid does not inhibit SARS-CoV-2, but it may bind to SARS-CoV-2 3CLpro and effectively impede the binding of SARS-CoV-2 -S1 protein with ACE2 (IC_50_ 56.06 μg/mL) and thus have the potential to block SARS-CoV-2 infection. Serrano-Aroca et al. (2021) advocated the use of alginate-based biomaterials for the management of COVID-19 [207]. Indeed, these biomaterials exhibited antiviral activity against a wide range of viruses, including the HIV-1 [208,209,210], hepatitis A, B, and C viruses [210,211,212,213], Sindbis virus [213], RABV [214], rubella virus [215], influenza virus [216], HSV-1 and 2 [74,76,77,101,213,217], poliovirus type 1 [213,218], potato virus X [219], tobacco mosaic virus [220,221], and murine norovirus [211,212]. Furthermore, the toxicity of these elements has been determined to be very low or non-existent. The antiviral mode of action is mostly attributed to viral aggregation and inhibition caused by interactions between alginate-based materials and viral envelope components. Alike SARS-CoV-2 many of these viruses are enveloped positive-sense single-stranded RNA viruses, making alginate-based materials extremely promising in the COVID-19 pandemic. Alongside the above-mentioned sulfated polysaccharides from marine origin, sulfated rhamnan and sulfated glucuronorhamnan also have antiviral activity against EV71 and IV [103,222,223].

### 3.2. Animal-Derived Compounds

#### 3.2.1. Heparin

The linear-structured, sulfated polysaccharide heparin is made up of repeating sequences of a uronic acid and D-glucosamine residues, and both of them are joined by 1,4-glycosidic linkages. The uronic acid can be β-D-GlcA or its C-5 epimer, α-L-IduA. Iduronate can be *O*-sulfated at position-2, whereas glucosamine can be N-sulfated, N-acetylated, or unmodified, and decorated with *O*-sulfates at position-6 and, less frequently, at position-3 [224,225]. This glycosaminoglycan (GAG) shows broad-spectrum activity against enveloped viruses including coronaviruses [110,226], SARS-CoV-2 [109,227,228,229,230,231,232,233], SARS-CoV-19 [111], zika virus [234], enterovirus 71 [235], echovirus [236]. In 2020, Mycroft-West and co-workers demonstrated that heparin inhibits SARS-CoV-2 infection in vitro [237]. The high incidence of thromboembolic events in COVID-19 patients suggests that coagulopathy plays an important role in the SARS-CoV-2 pathogenesis [39]. This already makes the anticoagulant molecule heparin a unique, potentially curative agent that appears to be a powerful, readily available measure to address the ongoing crisis associated with COVID-19 disease. This GAG also exhibits anti-inflammatory activity [238]. The antiviral, anticoagulant and anti-inflammatory activity of heparin against SARS-CoV-2 form a unique therapeutic combination [111]. Thus, repurposing heparin mimicking molecules such as sulfated polysaccharides to fight COVID-19 appears to be a powerful, readily available measure to address the current pandemic. Since many viruses employ cell-surface HS for attachment, it’s an attractive broad-spectrum antiviral target [72]. The first step in the cascade of interactions required for viral attachment is often the binding of a viral protein to HS [239]. For the reason that HS and heparin share similar glycosyl building blocks, and HS-binding proteins interact with heparin as well, heparin is drawing attention in COVID-19 treatment beyond its anticoagulant capabilities. Recently, both heparin and HS have been found to attach to S1 RBD [40,237,240], encourage a conformational change in SARS-CoV-2′s S1 RBD [237], and hinder SARS-CoV-2′s cellular invasion [41]. This drug has also been demonstrated to stop the binding of the SARS-CoV-2 spike protein to a human cell line [232], as well as the entry of pseudoviruses expressing the SARS-CoV-2 spike protein into human cells [109]. The heparin-derived drug enoxaparin also inhibits pseudovirus entrance [109], and hence LMWHs may be useful in COVID-19. Besides, LMWH treatment of COVID-19 patients was found to considerably reduce plasma levels of IL-6, a critical cytokine linked to the disease’s immunopathogenesis, in a retrospective clinical investigation [241].

#### 3.2.2. Chondroitin Sulfate

Chondroitin sulfate (CS) is a linear polysaccharide made up of repeated units of (1,4)-β-glucuronic acid (GlcA) and (1,3)-β-N-acetyl-galactosamine (GalNAc) that have sulfate groups at various places [242,243,244,245]. Based on sulfate position, CS has been dissented into four subtypes: CS-A, CS-C, CS-D, and CS-E. CS type A (CS-A) comprises GalNAc units sulfated at C-4, whereas CS-C has C-6 sulfated GalNAc units. The C-2 sulfated GlcA and C-6 sulfated GalNAc units make up Chondroitin sulfate type D (CS-D). GalNAc residues disulfated at C-4 and C-6 positions make up chondroitin sulfate E (CS-E). Marchetti et al. (2004) found that CS types A, B, C, and D had modest antiherpetic action [246], whereas CS-E isolated from squid cartilage had significant antiviral activity [247]. Antiviral activity of CS-E was seen against DENV [115]. And that CS-E showed antiviral activity as an entry inhibitor targeting the E protein of DENV. As reported by Kato and coworkers [115], shared carbohydrate determinants on CS-E may be key epitopes for DENV interaction and may be responsible for DENV inhibition. Studies on the structure-function correlation of CS in different biological systems have been hampered by its structural intricacy. Enzyme-based CS oligosaccharide syntheses have recently emerged as promising strategies for producing structurally specified oligosaccharides [248]. Polysaccharides with structures similar to GAGs, isolated from marine invertebrate species, have antiviral activity with a low anticoagulant potential [249]. Lian and colleagues (2013) discovered that a fucosylated GAG from an echinoderm has considerable anti HIV-1 efficacy [250]. The mechanism involves targeting CD4i of gp120, which results in HIV-1 entry inhibition. Another study [113] found that fucosylated chondroitin sulfate (FCS) derived from the sea cucumber *Thelenota ananas* had anti-HIV action, inhibiting numerous strains of HIV-1 reproduction with varying potencies. This polymer (FCS) can bind potently to recombinant HIV-1 gp120 protein, but it does not block recombinant HIV-1 reverse transcriptase. Thus, several polysaccharides from marine algae, and animal sources displayed effective inhibitory effects against a number of human and animal viruses.

Table 1 includes additional data on chemical features and antiviral activities of naturally occurring sulfated polysaccharides [71,73,75,83,84,88,96,97,98,99,102,112,114].

## 4. Sulfated Polysaccharides Generated by Chemical Sulfation Reaction

In 1987 it was observed that sulfated polysaccharides synthesized by a chemical sulfation reaction on polysaccharides are capable of inhibiting HIV [70]. Based on earlier research from the 1960s, dextran sulfate, the chemically sulfated derivative of an α-1,6-linked glucan namely dextran, was reported as a strong inhibitor of HIV with a 50% effective concentration at 0.1 μg mL^–1^ [70,251]. Afterwards, researchers began investigating a wide variety of other synthetic sulfated polysaccharides, and the results were promising (Table 2) [31,43,44,45,46,47,96,252,253,254,255,256,257,258,259,260,261,262,263,264,265,266,267,268,269,270,271,272,273,274,275,276,277,278,279,280]. Notably, these polysaccharides’ action spectrum has been displayed to comprise different enveloped viruses, encompassing viruses that appear as cunning infectious agents such as HSV and HCMV in immunocompromised patients [31]. Sulfated dextran, one of the few polymers that moved to clinical trials, hit multiple problems. The polymer had a reduced lifetime, quite low bioavailability, limited central nervous system penetration, and undesirable side effects. The most serious disadvantage was that it significantly raised circulating levels of the p24 antigen, implying that the polymer aided HIV multiplication [281]. Curdlan sulfate, another sulfated glucan containing a backbone of β-1,3-linked Glc*p* residues, synthesized by the chemical sulfation reaction of curdlan, a bacterial polysaccharide, efficiently prevents entry/fusion and restricts antibody-dependent enhancement of DENV infection in vitro [257]. A number of sulfated polysaccharides synthesized from plant structural polysaccharides such as cellulose, hemicelluloses and pectin, and gum polysaccharides also displayed potent antiviral activities against different viruses. In a comparative evaluation of sulfated galactomannan synthesized from diverse sources such as fenugreek gum, guar gum, tara gum, and locust bean gum, Muschin et al. (2016) found that these polymers have prominent anti-HIV and anti-DENV activities (Table 2) [262]. Electrostatic interaction of negatively charged sulfate groups of sulfated galactomannans and positively charged amino groups of surface proteins of viruses be the reason for these activities. Sulfated galactomannans generated from *Adenanthera pavonina, Caesalpinia ferrea*, and *Dimorphandra gardneriana* too exhibited activities against DENV-2 and PV-1 viruses in Vero cells [263,264]. The protection of sulfated konjac glucomannan against the HIV virus on lymphocyte MT-4 cells was detected using the MTT technique [268]. The EC_50_ (1.2–1.3 μg/mL) of this polymer is comparable to that of typical antiviral drugs. Cellulose sulfate derived synthetically by chemical sulfation of cellulose, the most abundant renewable polymer in nature is another polymer with potent anti-HIV activity [282,283,284]. In 2010, Saha et al., associated the inhibition of bovine herpes virus type-1 with sulfated derivatives of pectic polysaccharide fraction from the medicinal plant *Azadirachta indica* in HEp-2 cells [260]. Later on, Faccin-Galhardi et al. [259,261] demonstrated the inhibition of poliovirus and HSV-1 replication by these sulfated polymers, in similar conditions. Their antiviral effect originates due to the interference of polysaccharides at the early stages of HSV-1 replication. The chemically sulfated polysaccharide of *Angelica sinensis* had an antiviral effect on the mouse leukaemia virus, with polymer possessing the highest DS having the greatest antiviral effect [285]. Another study on polysaccharides from *Achyranthes bidentata* revealed that the sulfate functionality significantly enhanced virus clearance rates in swine reproductive and respiratory disease [286]. Likewise, the astragalus polysaccharide that had been chemically sulfated had a stronger activity against the infectious bursal disease virus than the native polymer [287]. Macroalgae are the primary source of non-animal sulfated polysaccharides in the marine environment. As shown in Table 2, chemical sulfation of both non-sulfated as well as sulfated polysaccharides such as lentinan [252,255], alginic acid [74,76,77,205], xylan [97], xylomannan [43,98], ulvan [45] and fucoidan [73,74,77] yielded derivatives possessing activity against TMV, HSV-1 and HSV-2. In general, the yield of sulfated polysaccharides obtained by the chemical sulfation reaction of polysaccharides varied from 17% to 118% depending on the nature of the reagents used for chemical sulfation and, also, on the nature of substate [18].

## 5. Synthesis of New Molecules Possessing Diverse Structures by a Single-Step Process Will Be a Useful Addition to the Arsenal of Antivirals

**Figure 1:**
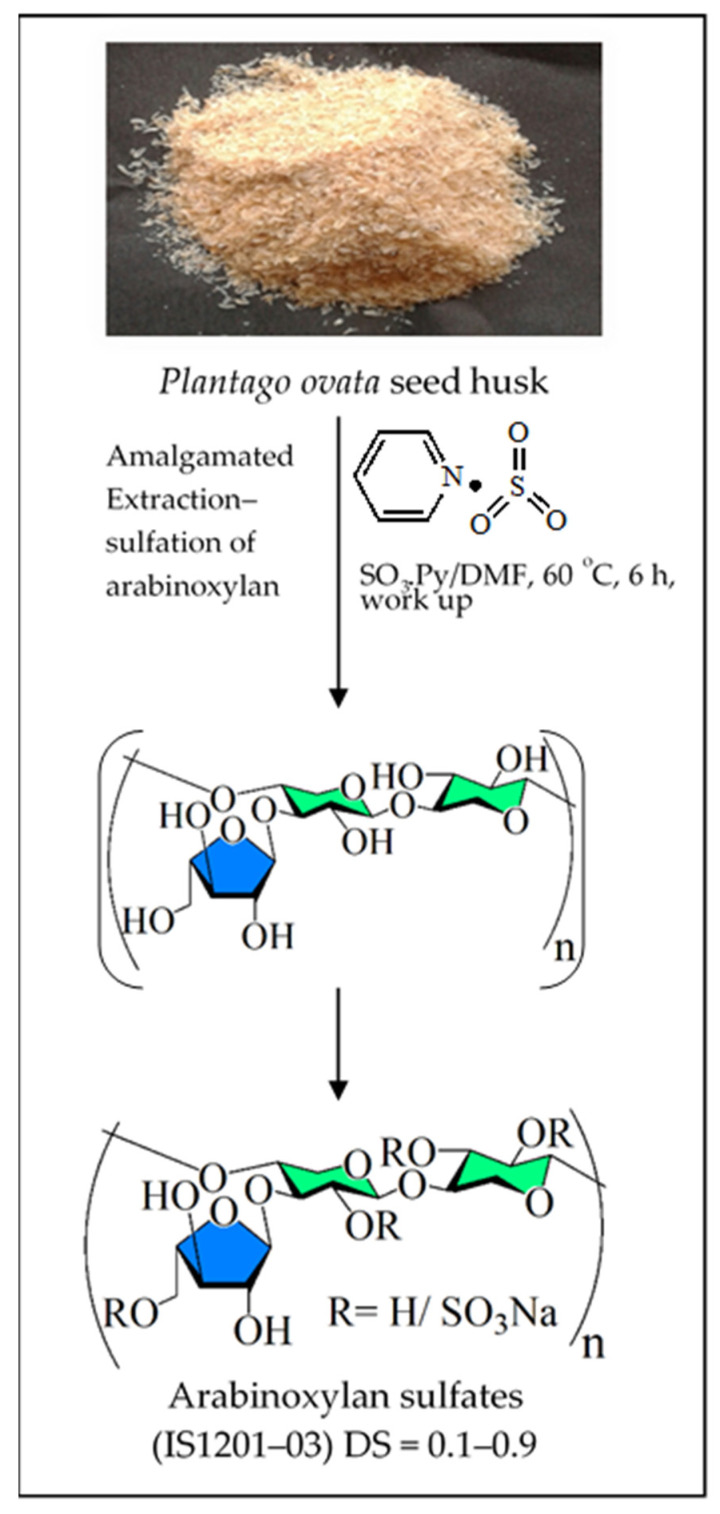
Schematic representation of the production of arabinoxylan sulfates from *P. ovata* seed husk using SO_3_.Pyr reagent in DMF at 60 °C [46]. Notably, drawings are not intended to be representative of the full sample composition.

Traditional chemical synthesis of sulfated polysaccharides is notoriously difficult, owing to the existence of a great number of stereocenters, the occurrence of alike functional groups, and the need to protect the glycosidic linkage patterns. Besides, fabricating polymers with reduced polydispersity adds to the difficulty. Chemical modification of the structure of naturally occurring molecules is an obvious way to change its properties. In fact, there are instances in the literature showing that chemical alterations can cause adverse or positive changes in the biological activity of natural compounds [11,288]. Recently, a unique one-step process that utilizes SO_3_·Pyr in dimethylformamide (DMF) solvent (SO_3_·Pyr/DMF) as reagent directly produces additionally sulfated xylomannan, starting from a seaweed namely, *Scinaia hatei* [43]. These synthesized polymers possess significant antiviral activities. The advantage of this process is that SO_3_·Pyr/DMF behaves as a “dual reagent”, as it effectively extracts polysaccharides from the plant material while promoting the chemical alteration of the hydroxy group existing in the polymer into sulfate functionality in the same pot as shown in Figure 1. Apropos methodological aspect, DMF as an aprotic polar solvent can extract polysaccharide, a polar compound containing hydroxyl and other polar functionalities. Moreover, this reagent also assists the dissolution process by destroying ionic and hydrogen bonds, which are found in the cell wall components of plant materials. Subsequent studies corroborate this economic procedure by creating various sulfated polysaccharides with different building blocks from plant materials [44,46,47,275]. The yield of sulfated polysaccharides obtained using the one-step procedure varies between 7% and 58% based on the starting material’s dry weight [46,47]. For this reason, the strategy has the capability to produce bioactive polymers through chemical diversification and functionalization of plant materials, which usually contain massive amounts of polysaccharides with different structures and functions. In particular, this simple procedure can eventually be standardized and made suitable for large-scale processes. It is expected that the outcome of this combined extraction-sulfation process will stimulate more research projects to apply this experimental method to produce biologically active compounds and possibly aim at a pharmaceutical development of plant-derived medicines.

## 6. Low Molecular Weight Heparin Mimetics

Heparin octasaccharides, synthesized from digested commercial heparin, serve as decoys for HSV binding; their structure varies from heparan sulfate in the degree of sulfation and MW, making them excellent mimics of the cell surface receptor. The decoys can then interact with HSV, most likely by binding to the glycoproteins gB or gC [289]. Based on recent data demonstrating that heparin oligosaccharides or comparable mimetics can reduce SARS-CoV-2 binding to target cells, the possibility of employing these oligosaccharides as COVID-19 therapeutic agents is gaining traction. In addition, non-coagulating heparin formulations that diminish cell binding and infectivity without inducing bleeding can be developed [40]. The potential of LMWH to mitigate cytokine storm in severe COVID-19 patients has also been investigated [241]. It has been observed that LMWH improves the coagulation dysfunction of COVID-19 patients and exerts anti-inflammatory effects by reducing IL-6 and increasing lymphocyte %. This group suggested that LMWH can be used as a potential therapeutic drug for the treatment of COVID-19, paving the way for a subsequent well-controlled clinical trial. Pixatimod (PG545), a clinical-stage heparan sulfate mimetic, is a potent inhibitor of a number of viruses, such as HSV-2 [290], HIV [291], RSV [292], Ross River, Barmah Forest, Asian CHIK, and chikungunya viruses [293], and DENV [294], with EC_50_′s ranging from 0.06 to 14 μg/mL. It has also been shown to possess virucidal activity, a unique feature only found in this particular class of amphiphilic HS mimetic [290,295]. In a prophylactic mouse HSV-2 genital infection model [290], a prophylactic Ross River virus mouse model [293], and a therapeutic DENV mouse model [294], in vivo efficacy of this compound was confirmed. Guimond et al. (2020) found that pixatimod binds directly to the SARS-CoV-2 spike protein S1 receptor-binding domain (RBD) and alters its conformation [296]. Notably, this site overlaps with the known ACE2 binding site in the S1 RBD. Furthermore, pixatimod inhibits the binding of recombinant S1 RBD to Vero cells which express the ACE2 receptor. Moreover, in assays with three different isolates of live SARS-CoV-2 virus, it was shown that pixatimod effectively inhibits viral infection of Vero cells. The neoagarohexaose (NA6), a 937 Da oligosaccharide derived from agarose by enzymatic hydrolysis, inhibited murine norovirus (MNV) replication with an EC_50_ of 1.5 μM in RAW264.7 cells. Moreover, it lowered viral RNA titer in a human hepatocellular carcinoma Huh7-derived cell line harboring a human norovirus subgenomic replicon. It was shown that IFN-β induction is the crucial pathway that is activated in a CD14-dependent manner by NA6 via the TLR4 to reduce norovirus loads in vitro and in mice. Chitooligosaccharides (COSs), prepared by chemical or enzymatic hydrolysis of chitosans, also have good biological activities including antibacterial activities [297]. The sulfated derivative of chitooligosaccharide (SCOS) possessed good anti-HIV activities at low MW (Mr 3000–5000) [298]. SCOS showed inhibitory actions on viral entry and virus-cell fusion via blocking the interaction between HIV-1 gp120 and CD4+ T cell surface receptors, suggesting that this marine-derived sulfated chitooligosaccharide has the potential to be developed into a novel antiviral agent. The neoagarohexaose (NA6), a 937 Da oligosaccharide derived from agarose by enzymatic hydrolysis, is a noncanonical Toll-like receptor 4 (TLR4) agonist with an EC_50_ of 1.5 μM in RAW264.7 cells against murine norovirus (MNV) [299]. This oligosaccharide also lowered viral RNA titer in a human hepatocellular carcinoma Huh7-derived cell line harboring a human norovirus subgenomic replicon. Although the exact molecular mechanism of NA6 recognition by the TLR4 complex remains to be elucidated, it was shown that IFN-β induction is the crucial pathway that is activated in a CD14-dependent manner by NA6 via the TLR4 to reduce norovirus loads in vitro and in mice. Low-molecular-weight mannogalactofucans (LMMGFs, <4000 g/mol) prepared by the enzymatic degradation of Undaria pinnatifida galactofucan (MF) were found to inhibit HSV type 1 with IC_50_ values of 2.64 and 2.42 μg/mL for LMMGFs and MF, respectively [300]. LMMGFs inhibited the viral entry on the host cell surface and also exhibited inhibitory activity directly against viral particles, as observed in a virucidal assay.

## 7. Relationship between Structures of Sulfated Polysaccharide and Their Antiviral Activities

**Figure 2:**
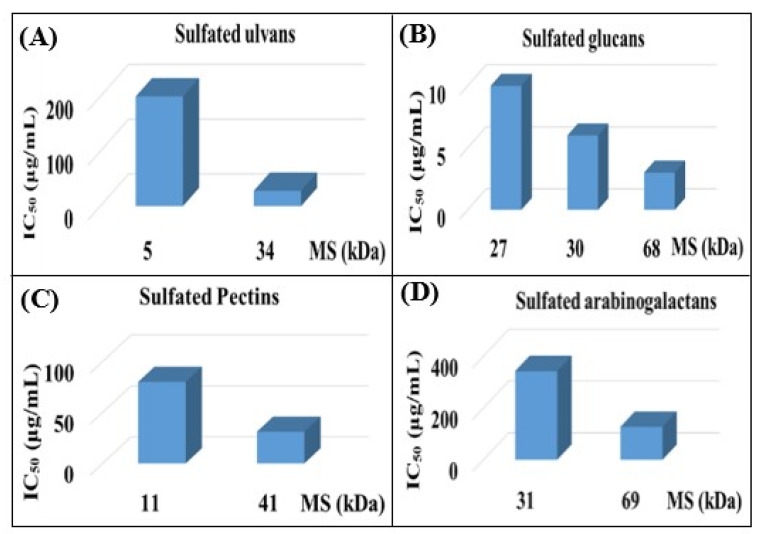
Comparison of antiviral activity of sulfated (**A**) ulvans against HSV-1 [45], (**B**) glucans against HCMV [47], (**C**) pectins against HSV-1 [259] and (**D**) arabinogalactans against HSV-1 [44], having different molecular masses (MSs). Antiviral activity was performed by plaque reduction assay in HEp-2 cells (human larynx epithelial cells carcinoma, ATCC CCL-23) (**A**,**C**), in Vero cells (ATCC CCL-81) (**D**) and by GFP-based replication assay in primary human fibroblasts (**B**).

In the past few decades, a substantial variety of sulfated polysaccharides with antiviral properties have been discovered (Table 1). In spite of this, due to significant dissimilarities in chemical structures of these biopolymers and a lack of data relating to both structure and function, establishing a compelling structure–activity relationship (SAR) was difficult. Additionally, depending on the types of viruses utilized, the potency of these biopolymers varied significantly in vitro (Table 1) reflecting differences in the interaction of these biopolymers with the different virus types. Even so, certain common structural motifs can be considered to be generally significant for antiviral activation, based on recent studies (Table 1). In the case of sulfated glucans, the crucial factor of antiviral activity is the molecular weight (MW). High MW is frequently associated with significant antiviral activity. For example, with dextran sulfates with MWs spanning from 1 to 500 kDa but the same sulfate content (81%), a significant increase in anti-viral activity was detected as the MW climbed from 1 to 10 kDa, even if antiviral activity tended to plateau at higher MS [31]. In a similar fashion, agarans [151], carrageenans [301], fucoidans [132] or chemically sulfated polysaccharides from *E. compressa* [45], Rice bran [47], *Azadirachta indica* leaves [259] and *Anogeissus latifolia* gum [44] show a comparable relationship (Figure 2). However, the general validity of this finding can be proven with sulfated polysaccharides of particular classes, such as ulvans, glucans, pectins, arabinogalactan or others, provided they show distinct structural similarities (Figure 2A–D). Sulfated polysaccharides of different classes with different degrees of sulfation, glycosyl composition and linkage pattern, branching pattern, have different structures and, therefore, dissimilar properties such as antiviral potencies. Regrettably, high-MS derivatives frequently have the drawback of lower tissue-penetrating properties, making them inapt for human use [302]. In contrast, oligosaccharides, such as those derived from carrageenan by chemical and enzymatic treatment, can have considerably higher bioavailability and biological activity [303]. An octasaccharide generated from a sulfated polymannuroguluronate (SPMG) could inhibit HIV adsorption [208] by targeting CD4 in lymphocytes [304]. According to new research [40], the administration of LMWH reduces mortality in individuals with severe coronavirus coagulopathy. They advocated using specially engineered heparan sulfate oligosaccharides as a new COVID-19 management method. Recently, polysaccharides were identified in buds of clove a potential natural anti-COVID-19 remedy [305].

**Figure 3:**
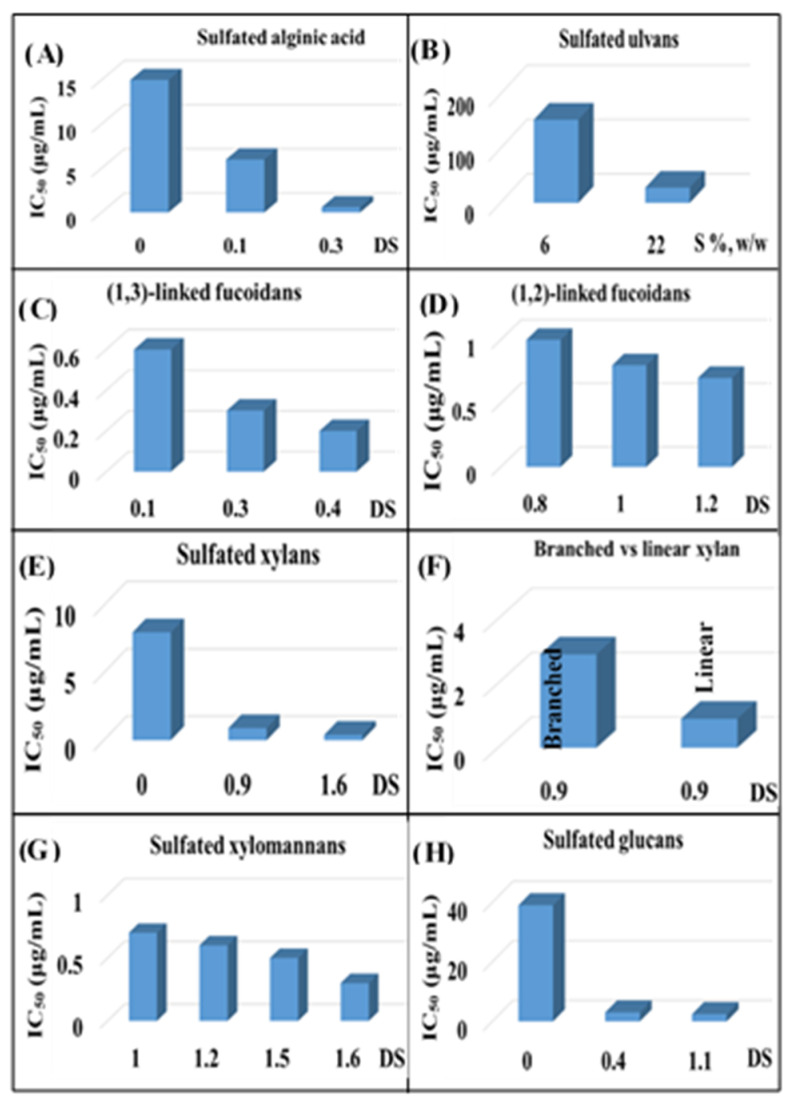
Comparison of antiviral activity of sulfated (**A**) alginic acids against HSV-1 [74,76], (**B**) ulvans against HSV-1 [45], (**C**) fucoidans against HSV-1 [77], (**D**) fucoidans against HSV-1 [73], (**E**) xylans against HSV-1 [97], (**F**) linear and branched β-1,4-xylans having same degrees of sulfation against HSV-1 [46,97], (**G**) xylomannans against HSV-1 [98] and (**H**) glucans against HCMV and HSV-1 [275] having different degrees of sulfation. Antiviral activity was performed by plaque reduction assay in RC-37 cells (African green monkey kidney cells) (**A**,**C**), in HEp-2 cells (human larynx epithelial cells carcinoma, ATCC CCL-23) (**B**), in Vero cells (**D**–**G**) and by GFP-based replication assay in primary human fibroblasts (**H**).

The sulfated polysaccharide’s degree of sulfation (DS) (i.e., number of sulfate groups per monosaccharide unit) is an especially important parameter that influences antiviral activity [29]. The significant positive relationship between DSs of naturally occurring sulfated polysaccharides, such as carrageenan [29], fucoidans [130,306], or chemically sulfated alginic acid [74,76], fucoidans [73,74,76,77], xylomannan [43,98], xylan [97], pectic polysaccharide [259,261], glucans [280], ulvans [45] and their antiviral activity (Figure 3), despite considerable structural differences, underscores the relevance of degree of sulfation. This is also true for chemically sulfated arabinoxylans, whose antiviral activities vary greatly depending on the degree of sulfation, with highly sulfated polysaccharides being more active [29,46,275]. Incidentally, a modeling study demonstrated that sulfated polysaccharides with a single sulfate or carboxylate group per monosaccharide unit failed to achieve stable binding with either S-protein receptor-binding domain (S-RBD) or ACE2, the human angiotensin-converting enzyme-2 [307] supporting the significance of the extent of sulfation on antiviral efficacy. Moreover, the removal of the sulfate functionality from ulvans [45], fucoidans [73], and chemically sulfated alginates [74,77], glucans [47,275], arabinoxylans [46], and arabinogalactans [44], drastically reduced antiviral activity confirming the importance of sulfate functionality. Sinha et al. (2010) demonstrated that the alginic acid of Sargassum tenerrimum possesses little anti-viral activity (IC_50_ of 15 μg/mL) [74], although the potency increased significantly (IC_50_ of 0.5 μg/mL) after the chemical sulfation reaction (Figure 3A). The alginate contains 1.0 carboxyl group per uronide residue and following the sulfation reaction the increase in charge density is less minuscule (1.0 carboxyl groups and 0.1 sulfate groups per residue). Thus, the antiviral activity of sulfated polysaccharide is not just a consequence of their high charge density, but also of the nature of anionic functionalities. Thus, sulfate functionality is vital for the antiviral activity of polymers, as opposed to the carboxyl group, which has little such effect. Incidentally, the extent of sulfation influences the strength with which heparin or HS fragments bind proteins [308].

**Figure 4:**
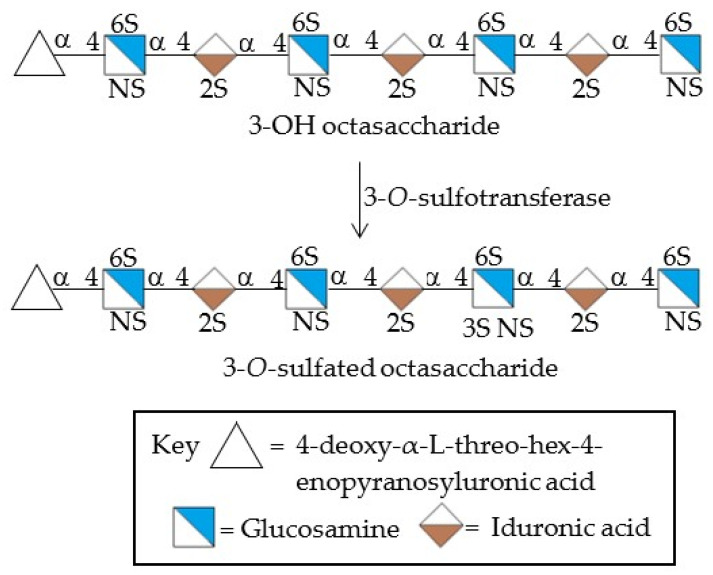
The preparation of 3-O-sulfated octasaccharide by 3-O-sulfotransferase. 2S, 2-O-sulfated; 3S, 3-O-sulfated; 6S, 6-O-sulfated; NS, N-sulfated. (Adapted from [310]).

The antiviral potency of sulfated polysaccharides depends, as well, on the precise position of the sulfate functionality. Initial investigators employed CS types A, B, C, and D but found little or no antiherpetic activity [246,309]. Later on, Bergefall et al. (2005) observed that CS-E isolated from squid cartilage possesses substantial antiviral activity [247] and it was explained by hypothesizing that the unique position of sulfates (at positions 4 and 6) in the main CS-E disaccharide unit is responsible for the antiviral activity. Similarly, Carlucci et al. (1997) found that the number of α-D-galactose 2,6-disulfate residues in natural carrageenan is closely connected to their antiherpetic activity, implying that the specific sulfation of galactose residues is important [301]. Copeland et al., found that a 3-O-sulfated octasaccharide made from heparin using an enzymatic method is more effective than a 3-OH octasaccharide in inhibiting HSV-1 infection [310]. Purified 3-O-sulfotransferase isoform 3 (3-OST-3) and a heparin-derived octasaccharide, namely 3-OH octasaccharide, were incubated to produce this octasaccharide (Figure 4). As a result, a precise sulfation pattern is required to prevent viral infection. Incidentally, the binding of heparin to different proteins is influenced by distinct heparin sulfation motifs [311]; some interactions require the unique 3-O-sulfate group, whereas most proteins use N- and 2-O-sulfates, which in heparin are extremely common [312]. Singh et al. (2015) also found that the pattern and extent of sulfation has a significant impact on the area on a protein wherever heparin fragments choose to bind, and that not all heparin fragments which bind have the same effect on the protein’s function [313].

The overall structural features of chemically sulfated polysaccharides have an impact on antiviral activity. Thus, sulfated glucans with no uronic acid and a high DS of 1.7 had higher antiviral activity than polysaccharides with high uronide content (9 percent, *w/w*) and a DS of 1.2 [275]. Likewise, sodium alginate (BEP) of *Laminaria angustata* had little anti-viral activity (IC_50_ of 25 μg/mL), yet a sulfated fucoidan (F2) had greater efficacy (IC_50_ of 0.65 μg/mL). This alginate possesses 1.0 carboxyl group per sugar unit, whereas the sulfated fucoidan is less anionic (0.05 carboxyl groups and 0.1 sulfate groups per sugar residue). Consequently, the antiviral activity of sulfated polysaccharides depends also of their structural specificities, including the composition of constituent saccharides. In particular, the effect of glycosidic linkages on viral inhibition is among the least studied polymer parameters. Linkage patterns and the anomeric configuration of the glycosidic bonds modify torsion angle values, and even minuscule variations in these angles can contribute to differences in the structure in solution. The more bending solution structure of heparan sulfate compared to heparin [314] demonstrates this, however, sulfation and glycosyl makeup changes may also play a role. Careful selection of the starting material is required to investigate the effect of anomers or linking patterns on the antiviral activity of a sulfated polysaccharide. In this regard, the fucoidan (F2) from *Padina tetrastromatica* with a DS of 0.8 has lower anti-HSV-1 activity (IC_50_ of 1.05 μg/mL) [73], whereas the fucoidan (S3) from *Laminaria angustata* with a lower DS value (0.4) has much stronger potency (IC_50_ of 0.2 μg/mL) [77]. Fraction S1 has 1,3-linked α-L-Fu*cp* residues in its backbone, whereas F2 fraction has 1,2- and 1,3-linked α-L-Fu*cp* residues in its backbone, indicating the relevance of glycosidic connections in anti-HSV-1 activity. Thus, fucoidan’s anti-HSV-1 activity is not only due to their DS, but also to their structural specificities such as glycosidic linkage pattern. Furthermore, linear β-1,3-xylan sulfates are more potent antiviral molecules than branched compounds with the same DS (Figure 3F). Advanced research is needed to justify the relevance of glycosidic linkages and branching on antiviral efficacy.

Chain conformation of polysaccharides is another structural feature that can influence biological activities. Generally, polymers can adopt various chain conformations such as random coil, duplex or triplex, rod-like, and sphere-like shapes, among others, in the solution [315]. Incidentally, polysaccharides with similar structural features, such as β-glucans, exhibit different bioactivities once the chain conformation is changed [316,317,318], suggesting that this structural feature greatly influences their biological activities [319]. Noticeably, the β-1,3-glucans with or without β-1,6-branched glucose residues adopt triple helices conformation in water but single random coils in DMSO or other polar solvents, associated with breaking/formation of hydrogen bonds. The broken triple helices can be reconstructed from the single chains, and triple helices can self-assemble into nanotubes with a hydrophobic cavity [315]. Relating to the dependence of antiviral activity on polysaccharides’ chain conformation almost no data is available. In 2006, Adhikari et al., advocated that to demonstrate inhibitory activity, the sulfate groups must be exposed to the macromolecule’s surface, which is highly dependent on the conformation and dynamic stereochemistry (a time and solvent dependent component of conformation) of the studied polysaccharide [71]. Therefore, further study on the solution properties and chain conformation of sulfated polysaccharides, as well as the effects of their conformation on antiviral activities is essential for the successful applications of these biopolymers. Combined, current data from our and other laboratories have shown that the antiviral properties of sulfated polysaccharides are dependent not only on charge density, and MW, yet also on the molecules’ structural features, such as glycosidic make up, linkage pattern, and chain conformation, which need to be analyzed in details in years to come. [44,45,47,259,275].

## 8. Antiviral Mode of Action (MoA) of Sulfated Polysaccharides

As an initial step of the viral replication cycle, precise attachment of a virus particle to its cell surface receptor is mandatory for viral entrance and ensuing intracellular multiplication [157,304,320]. As shown below with instances from the crucial HSV and HIV, virus polysaccharide interactions are also responsible for species and tissue tropism.

### 8.1. Sulfated Polysaccharides’ Role in Infections with Human Immunodeficiency Virus Type 1

Based on mechanism of action studies, it was found that sulfated polysaccharides exert their anti-HIV activity by interfering with the interaction between HIV’s glycoprotein gp120 and the CD4+ antigen receptor on T cells [31,251,321]. Initially, the specific mechanism of this connection was unknown. The V3 loop on the glycoprotein gp120 and the HIV-1 Tat protein were eventually identified as targets [322,323,324,325]. These materials also inhibit HIV-induced syncytium formation—the huge multinucleated cells produced by HIV to assemble, neutralize, and destroy T helper cells [112,322,323]. Furthermore, some of these polysaccharides may interact with HIV inside cells and impede replication by inhibiting reverse transcriptase (RT). Polysaccharides must be absorbed by infected cells in order for RT inhibition to occur, as demonstrated before with dextran sulfate and macrophages. Sulfated polysaccharides are hypothesized to function through two mechanisms: preventing viral adsorption and slowing reverse transcription [57,326]. Inhibition of viral adsorption and syncytium formation is currently thought to be the main mechanism [327].

### 8.2. The Putative MoA of Sulfated Polysaccharides in Infections with Herpes Simplex Viruses

HSV binds to heparan sulfate receptors in the course of the adsorption phase of viral infection, the phase when the virus adheres to susceptible cells via specialized receptors. It was realized that, unlike other GAGs (such as chondroitin sulfate) bound to cell surfaces, higher N-sulfation levels of heparan sulfate on cell surfaces could lead to viral binding [328,329]. HSV infection was resistant in cells that did not express heparan sulfate. Sulfated polysaccharides having antiviral action against HSV-1 and HSV-2 tend to be effective against both viruses with low cytotoxicity. Their inhibitory effects are mostly limited to the viral adsorption phase, where they interact either directly with the virus or with heparan sulfate on cell surfaces; adding these polymers to cell cultures after infection seldom results in significant viral suppression. Fucoidans derived from brown algae (Sargassum horneri), for example, showed no viral suppression when HSV or host cells were pre-treated with the sulfated fucoidan [330]. Their antiviral activity was observed only during viral infection, implying that the polysaccharide may interact with other membrane molecules on host cells (i.e., not heparan sulfate) while still interfering with virus–cell fusion [330]. As previously stated, the MW of these polysaccharides has a significant impact: high-molecular-weight polysaccharides are more effective at inhibiting HSV-1 and HSV-2. Desulfated polysaccharides were similarly shown to have less inhibition when added to HSV-infected cell cultures [83]. Other mechanisms of action have been investigated, including stimulating B cell and cytotoxic T lymphocyte production (sulfated fucoidan) or interfering with DNA replication, transcription, and viral protein production. In some cases, the polysaccharides showed broad antiviral activity against a variety of HSV-1 strains [331]. Heparin octasaccharides, made from digested commercial heparin, act as decoys for HSV binding; their structure differs only in degree of sulfation from heparan sulfate, making them ideal cell surface receptor mimics. The decoys can then interact with HSV by binding to the glycoproteins gB or gC, which is most likely the case [289].

### 8.3. The Putative MoA of Sulfated Polysaccharides in Infections with SARS-CoV-2

Cellular heparan sulfate (HS) has been found to bind SARS-CoV-2 spike glycoprotein (SGP) and co-operate with cell surface receptor angiotensin-converting enzyme 2 (ACE2) to mediate SARS-CoV-2 infection of host cells [332,333,334,335]. The SARS-CoV-2 SGP has a major role in the early infection process, where the S1 domain enables the binding and the S2 domain mediates the engulfment of the virions by membrane fusion [336]. Investigation of the SARS-CoV-2 SGP sequence exposed the furin-like cleavage site at GAG-binding motif resides within S1/S2 proteolytic cleavage motif [332,337,338]. Interestingly, the presence of a furin cleavage site at the S1/S2 boundary of SARS-CoV-2, in contrast to SARS-CoV-1, which does not have such a cleavage site, is implicated as the cause of increased infectivity of SARS-CoV-2 [338]. Sulfated polysaccharides are thought to act in a similar fashion on SARS-CoV-2 as against other enveloped viruses, i.e., the inhibition of virus adsorption, virus internalization and uncoating. However, other modes of action have also been described, such as the inhibition of 3CL^pro^ protease by the phlorotannin Dieckol [339]. Sulfated polysaccharides may also inhibit the expression and activation of epidermal growth factor receptors, which have inhibitory effects on coronaviruses [340].

### 8.4. Additional Aspects of MoA That are Independent of Virus Entry

The inhibition of viral entry into cells, as mediated by numerous natural source-derived compounds including sulfated polysaccharides, is based on the interaction of these compounds with cell surfaces or viral glycoproteins/virion proteins or both. It should be taken into account, however, that many cell surface-related processes like ligand binding, cell-to-cell contacts, or drug interaction are also translating into intracellular responses. A basic principle of the signaling pathways involves the cell surface stimuli that are transduced by membrane regulators, such as receptor signaling kinases, towards intracellular regulation, specifically also into the cell nucleus thus typically resulting in a modulation of transcription activity of individual genes. Given this background, it appears quite plausible that surface-active antiviral substances may either induce additional intracellular responses or even specifically exert an MoA that is independent from the inhibition of viral entry. Recently, we reported on examples of such intracellular modulation effects in the case of cytomgalovirus replication inhibited by natural source-derived sulfated glucans [47]. In this context, we demonstrated that the antiviral MoA was not exclusively based on the inhibition of viral entry, but was also linked to additional later effects of intracellular replication. When the time of addition of the compounds was varied, experimental data clearly indicated that the main anti-HCMV activity occurred at the stage of viral entry. Interestingly, however, we also noticed that after the viral entry phase, an increased antiviral effect was measured when the compounds continuously remained on the cells, including the viral post adsorption period, in which virus replication was maintained for several days. This additional effect of long-term treatment either could be due to inhibition of the second or third round of virus replication or, alternatively, could indicate an additional intracellular effect of the glucans bound to the cell surface or to virions. This latter point was addressed by measuring the expression levels of viral proteins belonging to immediate-early, early, and late markers of the viral replication cycle. Indeed, a partial block of protein synthesis was detected at the early stages of viral replication even when the substances were applied after virus infection. Furthermore, as a clear-cut result, viral protein expression was already blocked at the immediate early level of gene expression when substances were added in a mode of drug preincubation-adsorption-postinfection. Subsequently, the production of viral early and late proteins was consequently also inhibited, while the addition of substances later than virus entry did not have a marked effect. This indicates that some additional postentry effects of cell surface-bound sulfated glucans limited the efficacy of intracellular viral replication. Theoretically, such intracellular may also contribute to the viral modulation of innate immune responses, although this aspect of MoA awaits further investigation. As an additional point to be addressed for these types of compounds is the question of whether the antiviral potency of sulfated polysaccharides is dependent on specific cell types or viral tissue tropism. Principally, it appears plausible that some specificity may be directed to individual cell types, especially in these cases when limited quantities of viral entry receptors are expressed on individual cells. Then, it is feasible to expect a more pronounced entry-inhibitory efficacy of these compounds, however, detailed experimental proof of this concept is still missing. The current understanding is that most of the sulfated polysaccharides with antiviral activity are relatively broad-acting as based on pleiotropic principles.

## 9. Polysaccharide-Based Compounds Possess an Intrinsic Potential of Broad-Spectrum Antiviral Activity

Hundreds of viruses cause disease in humans, yet there is no specific medication for the majority of them [3]. The fact that novel viruses frequently emerge among humans as a result of spillover from other regions of the animal kingdom adds to the difficulty [4,5]. The scarcity of antiviral drugs that can be quickly mobilized and deployed for the treatment of re-emerging or new viral illnesses was highlighted by the SARS-CoV-2 pandemic. Broad-spectrum antivirals are one way to get around this problem. Polysaccharides are ideal for this purpose since polysaccharide-based antivirals tend to prevent the physical attachment and entry of the viral particles (Table 1 and Table 2). Thus, polysaccharides that attach to one virus and prevent cell attachment may be able to block viruses possessing comparable cell attachment mechanisms. Examples of broader acting antiviral activities of a natural source-based compound, not limited to one virus species alone, have been demonstrated by our group before [47,275,341]. Table 1 displays various polysaccharides that are able to inhibit significantly an array of viruses including SARS-CoV-2. Thus, it may be assumed that sulfated polysaccharides to be generated from natural products by using SO_3_.Pyr or Oleum-DMF reagent will exert broad-spectrum antiviral activities.

## 10. Polysaccharide-Based Antiviral Agents in Pre-Clinical and Clinical Studies

### 10.1. Carrageenan

Carrageenan is the most profoundly tested polysaccharide in the context of antiviral activity. It has been examined in humans for its ability to protect against sexually transmitted viruses such as HIV, HSV, and HPV, as well as RVs. Based on the finding that ι-carrageenan interferes directly with HPV adsorption to human sperm cells, two trials have been conducted, both of which have shown that carrageenan-based gels are effective against HPV transmission [342] and are well tolerated [343]. McGill University (Canada) is now conducting a third experiment [344]. Additionally, seven clinical trials have been conducted since 1997 to prove the efficacy of a carrageenan-based gel (carraguard) as a vaginal microbicide against HIV and HSV transmission. None of these studies, however, were able to conclusively demonstrate the efficacy of this topical treatment [120]. The ability of carrageenan to interfere with influenza virus type A adsorption to the host cell [94], as well as the discovery that a commercially available nasal spray containing carrageenan had good anti-IV type A activities in vivo using mice [94,345,346], has led to two clinical trials [166,347]. The results obtained demonstrated that direct local administration of ι-carrageenan with nasal sprays significantly reduced the duration of RV-associated cold symptoms. Carrageenan binds to viral glycoproteins, forming a physical barrier that prevents virions from infecting their target cells [121]. Fewer viruses were able to reproduce as limited viruses have access to epithelial tissue, resulting in lower viral titers and faster symptom relief. Notably, in the ι-carrageenan treatment group, the proinflammatory mediators FGF2, GCSF, IL8, IL1, IP10, IL10, and IFN2 were reduced [345]. Incidentally, the development of a nasal spray containing xylometazoline hydrochloride and ι-carrageenan for the symptomatic relief of nasal congestion caused by RVs is the most thriving case of utilization of carrageenan [348]. Several nasal sprays containing carrageenan are already available in Europe and Canada [349,350]. A clinical trial testing the preventive impact of a carrageenan nasal spray against SARS-CoV-2 is presently underway in the United States [351], and similar efforts to create an anti-SARS-CoV-2 carrageenan nasal spray are underway in the United Kingdom [352]. An overview of all currently conducted clinical trials against SARS-CoV-2 is shown in Table 3.

### 10.2. Fucoidans

Fucoidans have also been investigated in vivo using mice for their activity against different viruses [120]. Orally administered fucoidan from Undaria pinnatifida inhibited the propagation of avian IAV (subtypes H5N3 and H7N2) while increasing antibody production [353]. In a separate investigation, oral treatment of the same fucoidan to immunocompetent and immunocompromised mice infected with a lethal dosage of IV type A (subtypes H5N3 and H7N2) reduced virus replication, weight loss, and death in both groups while also lengthening their live expectancy. More intriguing was the fact that the use of fucoidan did not result in the development of drug resistance, which is usual when using traditional antiviral drug oseltamivir [354]. An intranasal application of fucoidan derived from Kjelmaniella crassifolia yielded the same results [143]. Fucoidan interacts with IAV surface enzyme neuraminidase (NA) in a host-independent manner to form a stable, inert complex that prevents viral entry into cells. Moreover, fucodians were shown to interfere with the activation of EGFR, PKCα, NF-κB and Akt, thereby inhibiting both IAV endocytosis and EGFR internalization [143]. More recently, Richards et al., demonstrated that the oral administration of fucoidan from Undaria pinnatifida was able to reduce symptoms and lung pathology after IAV infection [141], potentially by preventing virions from interacting with alimentary epithelia. In addition to infection and replication in the respiratory tract, influenza viruses are known to replicate in the alimentary tract [355]. Due to its binding and inactivation of IAV, orally administered fucoidan in the alimentary tract may prevent harmful interactions between the virus and alimentary epithelia. Richards et al., showed that the administration of fucoidan led to a minimal decrease in viral titers [141]. Intraperitoneal infusion (10 mg/kg) of fucoidans from Fucus evanescens protected mice from deadly intravaginal HSV-2 infection with a 50 percent efficacy [80]. Shikov and his colleagues recently published a study with more pharmacokinetic data on the antiviral activity of fucoidans [356]. Fucoidans may also exhibit indirect benefits as a randomized clinical trial with 70 volunteers revealed an increased immune response to seasonal influenza vaccination, when mekabu fucoidan was supplemented [357]. Further clinical trials should help to give more insights into the efficacy of fucodians against IAV infections.

### 10.3. Lectins

One of the most promising experimental drugs is griffithsin, a lectin extracted from red algae Griffithsia sp. Griffithsin binds asparagine-associated mannose structures in the case of HIV-1-infected cells, thereby inhibiting the binding of glycoprotein gp120 to its cell receptors [358,359,360,361,362,363,364]. Animal experiments demonstrated protection against high doses of the chimeric simian-human immunodeficiency virus (SHIV) in macaques and against vaginal HSV-2 and HPV pseudoviruses in mice [365,366]. A first clinical phase I study was completed in 2018 to evaluate safety, pharmacokinetics and pharmacodynamics of griffithsin in healthy women (NCT02875119). Another phase I study to determine the safety and pharmacokinetics of a griffithsin enema was conducted in 2019, however was prematurely terminated due to the COVID-19 pandemic [367].

### 10.4. Spirulan

The sulfated polysaccharide spirulan, existing as an ionic calcium or sodium form, has been isolated from Arthrospira platensis [368]. This polymer proved effective against enveloped viruses, including HSV-1, mumps virus, MV, HCMV, IAV and HIV-1 [368]. The very promising potential of calcium spirulan against HSV infections has been demonstrated by a recent study [369]. The clinical trial performed on 198 volunteers clearly illustrated a prophylactic effect of calcium spirulan. Furthermore, the topical application of a microalgae extract containing spirulan, as analyzed against herpes labialis, was superior to topical acyclovir [120]. Mechanistic analysis inficated that calcium spirulan blocks the attachment and penetration of HSV-1 into mammalian epithelial cells with a potency that proved to be at least comparable to acyclovir. In addition, an inhibitory effect onto the cellular entry of Kaposi sarcoma-associated herpesvirus/HHV-8 was also described [369].

### 10.5. Alginic Acids

The alginate-derived therapeutic agent, called 911, is a novel anti-HIV therapeutic agent that has been tested in phase 2 clinic trials [24]. This compound significantly limits the replication of HIV both in vitro and in vivo, and its activity is attributed to the inhibition of viral reverse transcriptase, the interference with viral adsorption, and the augmentation of immune function [370]. The sulfated polymannuroguluronate (SPMG) derived from alginic acid could inhibit HIV adsorption mainly through interfering with the interaction of virus gp120 protein with the CD4 molecule on the surface of T cells [208,304,371]. Incidentally, the octasaccharide unit was found to be the smallest active SPMG fragment capable of inhibiting syncytium formation [371].

### 10.6. Modified Polysaccharides

Antiviral characteristics and applications for several modified polysaccharides have also been initiated. Some sialic acid-modified polysaccharides, for example, have been produced with the intention of creating virus-capturing face masks or filters in the future [372,373]. Once carrageenan is replaced with the neuraminidase inhibitor zanamivir, two separate synergistic processes result in a polymer with improved inhibition [166]. Other changes, such as altering chitosan with disialooligosaccharide-terminated substituents, can improve polysaccharide antiviral activities [372]. Chemoenzymatically produced oligosaccharides have a higher inhibitory capacity than disialooligosaccharide monomers [372]. To understand the influence of these factors on inhibition, the degree of polymerization (DP) of chitosan and the degree of substitution (DS) of the disialooligosaccharide were changed in this work. Influenza inhibition increased as DP increased, while the inhibitory impact reduced as DS increased, perhaps due to steric crowding [372]. The fact that chitosan is positively charged and sialic acid is negatively charged at physiological pH complicates this work. As a result, bigger electrostatic complexes are likely to develop in these materials.

## 11. Future Perspective

The COVID-19 pandemic has highlighted the crucial need for antiviral chemicals that can be quickly deployed when a previously unknown or ignored virus suddenly becomes a worldwide emergency. Antiviral drug development for handling evolving viral illnesses would necessitate the consideration of compounds with novel modes of action. Polymeric chemicals lower the drug’s toxicity and the occurrence of side effects. They can also boost the efficacy of the real therapeutic ingredient. Because multivalent interactions are usually stronger than monovalent interactions, sulfated polysaccharides that are polyvalent can bind to multiple complementary receptors on biological targets at the same time. The most important aspect of polysaccharides is their structure, and the presence of functionality that can be altered by chemical reactions to induce and change biological activities. The extent of sulfation, MW, sugar composition, glycosidic linkage patterns, structures, and shape are all factors that determine the antiviral properties of polysaccharides. Consequently, these molecules should be investigated further as potential antiviral agents that can be used alone or in conjunction with existing medications. The majority of antiviral actions of sulfated polysaccharides have been extensively documented in vitro or in mice model systems. The next step would be to bring the most promising polysaccharides into clinical trials to investigate their activity in a controlled and randomized setting. The application of carrageenan as a nasal spray or oral drops as prophylaxis and early-stage treatment against common cold viruses and SARS-CoV-2 is thought to be a very promising aspect, which is currently evaluated by various clinical trials. The results of these investigations may help to establish the use of polysaccharides as a natural high-value antiviral drug with the advantage of a low rate of adverse effects.

## Figures and Tables

**Table 1:** Naturally occurring sulfated polysaccharides: molecular masses, sulfate contents (mol %) and their half-maximal inhibitory in vitro/effective concentrations (IC_50_, EC_50_) referring to the indicated viruses.

Entry and Compound	Origin	Molecular Mass ^a^	Sulfate (mole %)/DS	Analyzed Viruses	IC_50_/EC_50_ Value (μg/mL)	Comments on Antiviral Activity	References
**1. Fucoidan**	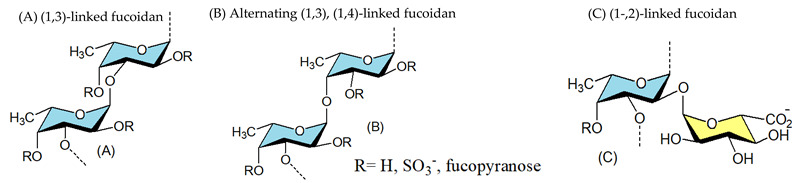 Adapted from [71] and [72]						
	*Padina tetrastromatica*	-	0	HSV-1 (HSV-2)	>100 (50)	Inhibition of viral adsorption to the cell, DS represents the key parameter	[73]
			0.8		1 (0.4)		
			1.0		0.8 (0.3)		
			1.2		0.7 (0.3)		
	*Sargassum* *tenerrimum*	30	2	HSV-1	1.4	Block of viral entry	[74]
		-	6		0.5		
	*Undaria* *pinnatifida*	9	10.4	HSV-1	2.5	Interference with virus-host cell binding, broad-spectrum activity	[75]
				HSV-2	2.6		
				HCMV	1.5		
				IV-A	15		
	*Sphacelaria indica*	-	0	HSV-1	N. D	Interfere with viral attachment and entry	[76]
		26	4		1.3		
		-	7		1.5		
	*Laminaria* *angustata*	56	4.2	HSV-1	0.6	Direct interaction with viral particles	[77]
		-	6.7		0.32		
		-	7.3		0.21		
	*Cystoseira indica*	-		HSV-1 (HSV-2)	35 (20)	Inhibition of viral attachment, desulfation removes antiviral effect	[78]
		-	8		2.1 (0.5)		
		35	0		>100		
		-	9		3 (1.3)		
	*Stoechospermm marginatum*	40	1–13	HSV-1 (HSV-2)	1.15–50 (0.78–50)	Interference with the HSV-1 replication cycle	[71]
	*Sargassum henslowianum*	66, 59	32, 32	HSV-1 (HSV-2)	0.9,0.5 (0.8,0.5)	Block of HSV-2 adsorption to the cell	[79]
	*Fucus* *evanescens*	-	-	HSV-1 (HSV-2){ECHO-1} [HIV-1]	80 (65) {110} [25]	Inhibition of the early stage of virus replication, broad-spectrum activity	[80]
		-	-		100 (85) {93} [25]		
	*Sargassum swartzii*	45	19.2	HIV-1	3.1	DS may be the key parameter	[81]
		30	24.5		1.6		
		>100	24		0.6		
	*Saccharina* *japonica*	100	-	SARS-CoV-2	8.3	Binding to the S protein of SARS-CoV-2	[82]
		12	-		16		
**2. Galactan sulfates**	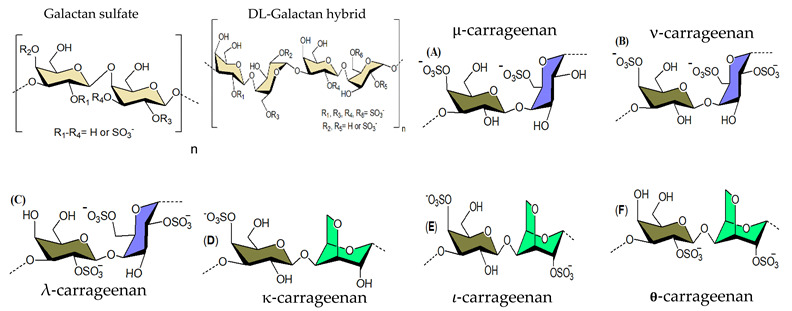						
	*Gracilaria* *corticata*	-	0	HSV-1 (HSV-2)	56 (24)	Inhibition of virus adsorption, interfering with the interaction of viral glycoproteins	[83]
		-	1		16 (8)		
		-	1.2		15 (7)		
		-	1.5		4(1.7)		
		-	0		>100 (37)		
		30	0.9		27 (14.6)		
		-	2		1.6 (1.1)		
		-	2.1		1.6 (1.5)		
	*Bostrychia* *montagnei*	6–46	11.2–24	HSV-1 (HSV-2)	13–50 (11–50)	Shielding off the positively charged sites	[84]
	*Gracilaria* *corticata*	165	11.6	HSV-1 (HSV-2)	0.19 (0.24)	Inhibition of virus entry by interaction with viral glycoprotein	[85]
		62	2.6		27.5 (38.5)		
		54	2.5		50 (45.9)		
	*Gymnogongrus torulosus*	18–77	-	HSV-1 (DEN-2)	0.6–16 (0.19–1.7)	Binding of the surface envelope glycoprotein	[86]
	*Schizymenia* *binderi*	380	22.2	HSV-1	0.76	Interference with the HSV–HS interaction	[87]
				HSV-2	0.63		
	*Solieria chordalis* (𝜄-carrageenan)	-	0.3–5.1	HSV-1	0.3–19	-	[88]
	*Meristiella gelidium*- 𝜄/Ϗ/ν carrageenan	-	29	HSV-2 (DENV-2)	0.06 (0.79)	-	[89]
		-	33		0.05 (0.14)		
		-	29		0.04 (0.21)		
	Ϗ, λ-carrageenan	-	-	HSV-1	3.7, 1.6	Inhibition of virus adsorption to the host cell, broad-spectrum activity	[90]
				HSV-2	2, 1.5		
				HIV-1	12, 1.9		
				CMV	2.8, 0.3		
				VSV	0.3, 0.2		
	𝜄-carrageenan	-	-	HSV-1	2	Inhibition of an undefined step in virus replication, broad-spectrum antiviral activity	[91]
				HSV-2	10		
				SFV	10		
				Vaccinia	10		
				ASF	10		
				EMC	10		
	κ-carrageenan	1–4	4–30	IAV	14.9–142	Inhibition of IAV multiplication	[92]
	ι-, λ-, κ-carrageenan	-	-	DENV-1 (DENV-2) {DENV-3} [DENV-4]	40.7 (0.4) {4.1} [8.2]	Inhibitors of DENV-2 and 3 multiplications in Vero and HepG2 cells, broad-spectrum activities	[93]
					>50 (0.1) {2} [4.2]		
					>50 (1.8) {6.3} [50]		
	ι- carrageenan	-	-	H3N2	0.04	Surface block of epithelia in IAV-infected animals	[94]
				H1N1	0.20		
	ι- carrageenan	-	32–39	IV-A, B	0.3–1.4	Inhibition of viral entry	[95]
				SARS- CoV-2	0.9		
**3. Xylomannan sulfate**	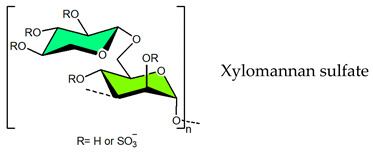						
	*Nemalion* *helminthoides*	14	19.4	HSV-1 (HSV-2) {DENV-2}	9.68 (3.72) {8.22}	DS may play an important role	[96]
		12	22.9		5.43 (2.79) {16.1}		
	*Scinaia* *hatei*	-	N. D	HSV-1 (HSV-2)	8 (12)	Mode of action directed to viral entry	[97]
		-	0.93		0.9 (0.4)		
		-	1.42		1.2 (0.22)		
		-	1.64		0.4(0.3)		
		-	1.95		1.4(0.4)		
	*Sebdenia* *polydactyla*	-	0	HSV-1	>10	DS may play an important role, DS of 1 is sufficient for antiviral activity	[98]
		150	0.6		2.8		
		-	1		0.7		
		-	1.2		0.6		
		-	1.5		0.47		
		-	1.6		0.35		
	*Scinaia* *hatei*	-	0	HSV-1 (HSV-2)	>100 (100)	Inhibition of virus-cell attachment	[99]
		160	8		0.5 (0.5)		
	*Scinaia* *hatei*	-	9	DENV-2	1.1	Interference with viral multiplication cycle	[100]
		160	8		0.6		
**4. Rhamnan sulfate**	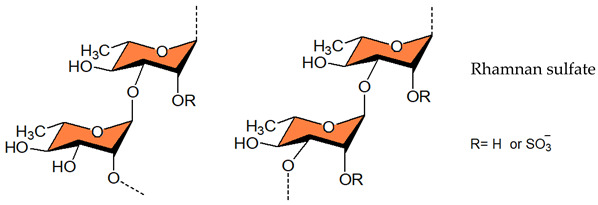						
	*Monostroma nitidum*	-	31.7	HSV-2	0.87	Inhibitor of HSV-2 entry	[101]
	*Monostroma Iatissimum*	-	-	HSV-1	0.78	Inhibition of virus adsorption, board-spectrum activity	[102]
				HCMV	1.7		
				HIV-1	1.5		
	*Monostroma* *latissimum*	513	26.1	EV71	-	Inhibition of viral replication	[103]
**5. Ulvan**	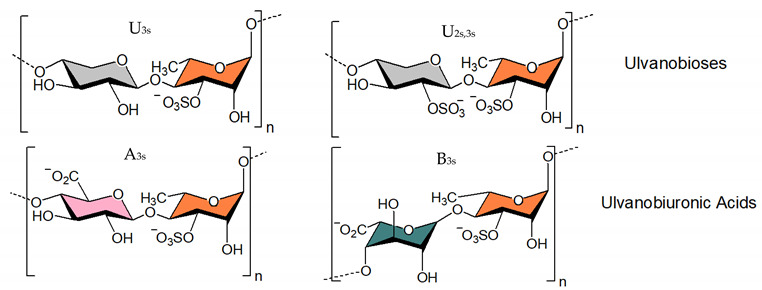						
						Adapted from [104]	
	*Ulva intestinalis*	-	-	MV	3.6	-	[105]
	*Ulva pertusa*	1068	17.7	VSV	-	Interaction with viral envelope glycoprotein	[106]
		39	17.9		0.6		
		18	18.1		15		
		5	17.1		6		
	*Ulva clathrata*	360	9.5	NDV	0.1	Inhibition of viral entry	[107]
	*Ulva armoricana*	-	-	HSV-1	373 321	Antiviral activity correlated to high levels of Rha*p*	[108]
**6. Alginic acid**	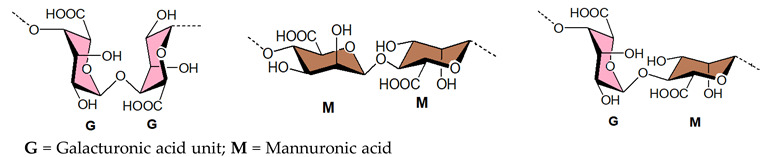						
	*Sphacelaria* *indica*	21	0	HSV-1	10	Interfere with viral attachment and entry	[76]
		-	8		0.65		
		-	9		0.6		
	*Sargassum* *tenerrimum*	26	0	HSV-1	15	Block of viral entry	[74]
		-	2		6		
**7. Heparin**	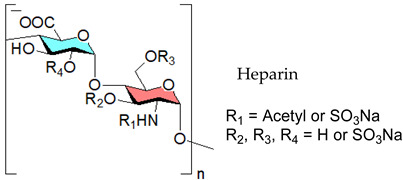						
	Heparin	-	15	SARS-CoV-2	5.99	Affinity to SGP	[109]
	Heparin	-	-	SARS-CoV	-	Protein binding responsible for SARS-CoV inhibition	[110]
	Heparin	-	-	SARS-CoV-2	-	Heparin may bind to viral protein	[111]
	Heparin	-	6.4	HIV-1 (HIV-2)	0.52 (1.7)	Block of virus adsorption	[112]
**8. Chondroitin sulfates types A, C, D and E (CS-A, CS-C, CS-D, CS-E)**	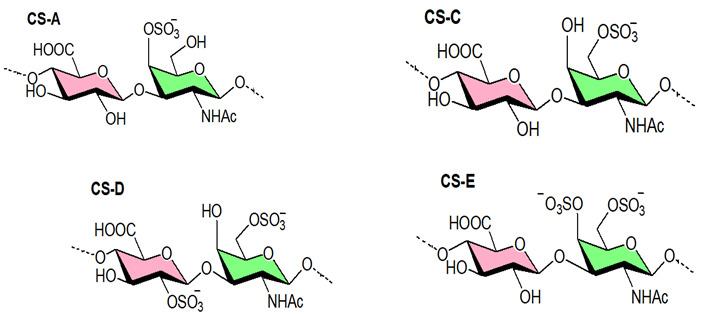						
	*Thelenota* *anana*	-	-	HIV-1	0.24–31.8	Potently binds viral gp120 protein	[113]
	Chondroitin sulfate	-	2.41	HSV-2	74.8	-	[114]
		-	-		26.6		
	Chondroitin sulfate	-	-	DENV-1	0.53	Entry inhibitor targeting viral E protein, broad spectrum activity	[115]
				DENV-2	3.80		
				DENV-3	1.38		
				DENV-4	0.30		
				JEV	0.93		

^a^ Molecular weight values are rounded off; - no data found. Notably, drawings are not intended to be representative of the full sample composition. EC_50_, half-maximal inhibitory compound concentration measured by eukaryotic cell-based assays. IC_50_, half-maximal inhibitory compound concentration measured by in vitro enzymatic assays.

**Table 2:** Sulfated polysaccharides obtained by chemical sulfation reaction on isolated material: molecular weights, sulfate contents (mol %) and their half-maximal inhibitory *in vitro*/effective concentrations (IC_50_, EC_50_) referring to the indicated viruses.

Entry and Compound	Origin/Preparation Notes	Molecular Mass	Sulfate (mol %)/DS	Analyzed Viruses	IC_50_, EC_50_ Value (μg mL^−1^)	Comments on Antiviral Activity	References
**Chemically sulfated plant/fungal/bacterial polysaccharides**							
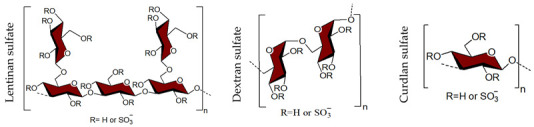							
**1. Lentinan sulfate**	*Lentinus edodes* in HSO₃Cl-Py	-	0	TMV	-	Affinity of the polyanion towards positive ions on viral particles	[252]
		-	0.69		-		
		-	0.98		-		
		-	1.37		-		
	*Lentinus edodes* In HSO₃Cl-Py	-	0	TMV	-	Affinity towards TMV coat protein	[253]
		-	0.98		-		
	*Lentinula edodes* In HSO₃Cl-Py	-	0	NDV	-		[254]
		-	0.69		-		
		-	0.98		-		
		-	1.37		-		
	*Lentinus edodes* In HSO₃Cl-Py	-	0	IBV	-	Activity refers to DS (up to DS value 0.98)	[255]
		-	0.69		-		
		-	0.98		-		
		-	1.37		-		
**2. Dextran sulfate**	*Leuconostocmesenteroid* In HSO₃Cl-Py	1–70	81	HIV-1 HIV-2	0.2–7.1 0.1–3.9	Activity by shielding off the positively charged sites in the V3 loop of the viral envelope glycoprotein gp120	[31]
**3. Curdlan sulfate**	Curdlan in DMSO In SO_3_-py	6.2–10.8	0.66–1.55	HIV	0.04–0.4	DS is an antiviral determinant, but not the position of sulfate groups	[256]
	Curdlan In SO_3_-py	14	1.4	DENV-2	0.26	Inhibition of viral infection at the step of virus-host cell binding	[257]
		6	1.5		0.37		
	Curdlan In SO_3_-py	172	9.23	HBV	-	Interference with virus binding to host cells surfaces	[258]
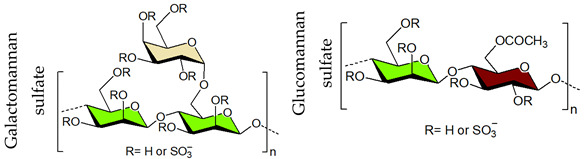					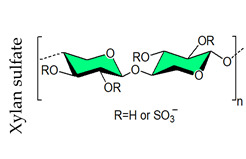		
**4. Pectin sulfate**	*Azadirachta indica* In SO_3_-pyridine	41	4	HSV-1	31.1	Interference at an early stage of the viral replication cycle	[259]
		11	4		80.5		
		80	-	BoHV-1	32.1	Inhibition of virus-cell adsorption	[260]
		41	4		105.2		
		41	4	PV-1	37.5	Inhibition of the initial stage of viral replication	[261]
		11	4		12.1		
**5. Galactomannan sulfates**	Fenugreek gum In PSA and SO_3_-Py	7–24	0.7–1.4	HIV	0.4–1.6	Electrostatic interaction between negatively charged sulfate groups and positively charged amino groups of viral surface proteins	[262]
	Guar gum In PSA and SO_3_-Py	8–23	1.1–1.3		0.3–0.6		
	Locust bean gum In PSA and SO_3_-P	9–23	1–1.4		0.3–0.7		
	Tara gum In PSA, SO_3_-Py	6–24	0.7–1.3		0.2–8		
	*Adenanthera pavonina* In HSO₃Cl-Py	700	1.21	PV-1	1.18	Inhibition mainly the initial stages of viral infection	[263]
	*A. pavonina* *C. ferrea* *D. gardneriana* In HSO₃Cl-Py	-	0.72–0.82	DENV -2	-	Entry inhibitor of DENV-2	[264]
	*Mimosa scabrella* In HSO₃Cl-Py	-	0	HSV-1	n.a.	Inhibition of the virus attachment step	[265]
		620	0.62		<2.5		
	*Leucaena* *leucocephala* In HSO₃Cl-Py	-	0	YFV	n.a.	Block of early stages of viral replication	[266]
		574	0.50		200		
	Commercial Galactomannans In PSA	4	1.11	HIV	2.14	Electrostatic interaction between sulfate and amino groups	[267]
		4.6	1.12		1.93		
		5.2	1.15		0.44		
		6.5	1.16		0.23		
		7.5	1.52		0.18		
**6. Glucomannan sulfate**	Konjac glucomanna In PSA and SO_3_-Py	8	1.3	HIV	1.4	Electrostatic interaction between sulfate and amino groups	[268]
		8	1.4		1.3		
		8	1.9		1.6		
		56	1.6		0.7		
	Konjac glucomannan In HSO₃Cl-Py	-	33.11	CVB	148	Block of virus invading function	[269]
	*Agaricus brasiliensis* In HSO₃Cl-Py	86	14.77	HSV-1 HSV-2(vivo)	17.27 4.73	Inhibition of viral attachment and entry	[270]
	*Agaricus brasiliensis* In HSO₃Cl-Py	86	14.77	HSV-1 HSV-2	1.24 0.39	Inhibition of viral attachment	[271]
**7. Xylan** **sulfate**	*Scinaia hatei* In SO_3_-py		0–1.95	HSV-1 HSV-2	0.4–7.6 0.22–11.7	Inhibition of viral entry	[97]
**8. Ophiopogon polysaccharide**	*Ophiopogon japonicus* In HSO₃Cl-Py		0.83–1.52	NDV	-	Inhibition of viral adsorption	[272]
**9. Glycosa minoglycan**	*Pseudomonas* In H_2_SO_4_-DMF	-	-	IVA	>100	Inhibition of viral attachment to the cell prior to viral penetration	[273]
		130	4.3		16		
		150	8		5.2		
**Sulfated polysaccharide generated by combined extraction-sulfation from natural sources**							
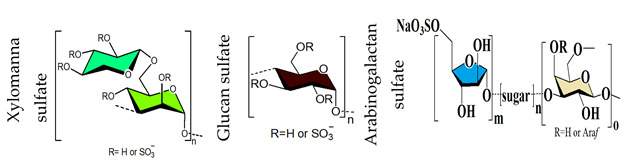						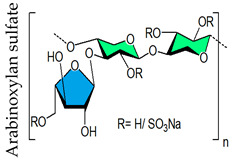	
**10. Xylomannan sulfate**	*Scinaia hatei* In SO_3_⋅Py	12–74	11.3–50.1	HSV-1 HSV-2	0.67–88 (0.22–38.55)	Sulfate groups represent hallmark of activity	[43]
**11. Glucan sulfate**	Rice bran In Oleum-DMF	68	1.6	HSV-1 GPCMV MCMV HCMV	3–>10 8.1–>10 3.4–8.1 2.4–6.5	Inhibition of viral entry	[47]
		30.5	1.7				
		27.3	1.2				
	Rice bran In SO_3_-Py	-	0.3–0.4	HCMV	n.a.	Sulfate groups represent hallmark of activity	[274]
		-	2		3.46		
	*Eleocharis dulcis* fruit In oleum–DMF	-	0	HCMV	>30	Mode of antiviral action mostly based on the inhibition of viral entry	[275]
		-	1.2		-		
		94	1.7		2.3		
		-	0.7		-		
	Oat Bran In HSO₃Cl-Py	500	0	HIV-1	n.a.	Negative compound charges bind to positively charged amino acids	[276]
		686	36.5		5.98		
	*Gastrodia elata* Bl In HSO₃Cl-Py	280	0	DENV-2	n.a.	DS is the determining factor of antiviral activity	[277]
		65	0.206		20.6		
		190	1.68		10.7		
	*Gastrodia elata* Bl In HSO₃Cl-Py	190	1.68	DENV-2	0.68	Interference with viral adsorption	[278]
	*Agaricus* *brasiliensis* In HSO₃Cl-Py	609	0	HSV-1 (HSV-2)	n.a. (n.a.)	Inhibition of viral adsorption and penetration	[279]
		127	1.88		6.7 (4.6)		
	Botryosphaeran In HSO₃Cl-Py	-	0	HSV DENV	39.3 or n.a.	Electrostatic interaction between sulfate and amino groups	[280]
		-	0.4		3.0 (66)		
		-	1.1		2.4 (78)		
**12.Sulfated Ulvan**	*Enteromorpha compressa* In Oleum-DMF	5	-	HSV-1	200	Electrostatic interference with the positive charge of viral glycoprotein	[45]
		34	22		28.2		
**13.Arabinogalactan sulfate**	*Anogeissus latifolia* gum In SO_3_⋅Py	-	0	HSV-1	n.a.	Inhibition of viral attachment and entry	[44]
		69	0.1		127		
		35	0.3		630		
		31	0.5		342		
**14.Arabinoxylan sulfate**	*Plantago ovata* seed husk In SO_3_⋅Py	31.3	0.1	HSV-1	n.a.	DS determines antiviral activity	[46]
		26.7	0.4		11.5		
		18.4	0.9		2.9		

n.a., no activity; CVB, Coxsackievirus B; PSA, piperidine-N-sulfonic acid; Py, pyridine; - no data found. Notably, drawings are not intended to be representative of the full sample composition. EC_50_, half-maximal inhibitory compound concentration measured by eukaryotic cell-based assays. IC_50_, half-maximal inhibitory compound concentration measured by in vitro enzymatic assays.

**Table 3:** Overview of all currently ongoing clinical trials of carrageenan against SARS-CoV-2 (listed at clinicaltrials.gov, accessed on 20 November 2021 ).

	Study Title	Identifier	Status	Results	Primary Outcome
1	Study to Investigate if Sucking a Coldamaris Lozenge Elutes Sufficient Iota-carrageenan to Inactivate Usual Common Cold Viruses	NCT04533906	Completed	Pending	Iota-carrageenan concentration in saliva
2	USEFULNESS of Topic Ivermectin and Carrageenan to Prevent Contagion of COVID 19 (IVERCAR)	NCT04425850	Completed	Published	Number of participants testing positive for COVID-19
3	Prophylaxis COVID-19 in Healthcare Agents by Intensive Treatment With Ivermectin and Iota-carrageenan (Ivercar-Tuc)	NCT04701710	Completed	Pending	Number of subjects who were diagnosed with COVID-19 in EG and CG
4	Carrageenan Nasal Spray for COVID-19 Prophylaxis	NCT04590365	Recruiting	Pending	Rate of COVID-19 infection
5	Efficacy of a Nasal Spray Containing Iota-Carrageenan in the Prophylaxis of COVID-19 Disease in Health Personnel Dedicated to Patients with COVID-19 Disease	NCT04521322	Recruiting	Pending	Diagnosis of COVID19 disease
6	Effect of Local Treatment(Carrageenan Nasal Spray and PVP-I Mouthwash) in Reducing Viral Load in Patients With COVID-19 (LT-COVID19)	NCT05049213	Recruiting	Pending	Change from baseline naso-pharyngeal viral load quantified by RT-PCR at Day 8
7	Prophylactic Treatment With Carragelose Nasal Spray to Prevent SARS-CoV-2, COVID-19, Infections in Health Care Workers	NCT04681001	Recruiting	Pending	Presence of COVID-19 symptoms including symptoms of respiratory viral infection
8	Efficacy and Safety Evaluation of Inhaleen Inhalation in Hospitalized COVID-19 Patients	NCT04793984	Recruiting	Pending	Clinical status of subjects as expressed on the WHO-8-Category ordinal scale

## Data Availability

The responsible authors declare that this article fully complies with the Data Availability Statements in section “MDPI Research Data Policies”.

## Abbreviations

AIV, avian influenza virus; CC_50_, half-maximal cytotoxic compound concentration; EC_50_, half-maximal inhibitory compound concentration measured by eukaryotic cell-based assays; IC_50_, half-maximal inhibitory compound concentration measured by in vitro enzymatic assays; DENV, dengue virus; GAG, glycosaminoglycan; HBV, hepatitis B virus; HCMV, human cytomegalovirus; HepG2, human hepatoblastoma cell line; HIV-1, human immunodeficiency virus type 1; HMPV, human metapneumo virus; HPV, human papilloma virus;; HSV-1, herpes simplex virus type 1; HSV-2, herpes simplex virus type 2;; IAV, influenza A virus; JEV, Japanese encephalitis virus; LMWH, low molecular weight heparin; MW, molecular weight; NDV, Newcastle disease virus; RABV, rabies virus; RV, Rhinovirus; SARS-CoV-2, severe acute respiratory syndrome coronavirus type 2; SI, selectivity index (CC_50_,EC_50_).
